# The long noncoding RNA ANRIL acts as an oncogene and contributes to paclitaxel resistance of lung adenocarcinoma A549 cells

**DOI:** 10.18632/oncotarget.16640

**Published:** 2017-03-29

**Authors:** Ran Xu, Yuqiang Mao, Kuanbing Chen, Wei He, Wenjun Shi, Yun Han

**Affiliations:** ^1^ Department of Thoracic Surgery, Shengjing Hospital, China Medical University, Shenyang, Liaoning, China

**Keywords:** long noncoding RNA, ANRIL, lung adenocarcinoma, chemotherapy resistance, paclitaxel

## Abstract

Long non-coding RNAs (lncRNAs) are a family of non-protein-coding RNAs that might affect Lung adenocarcinoma (LAD) chemo-resistance and most of them could be used as biomarkers and therapy targets. However, the potential function of lncRNA ANRIL contributed paclitaxel chemo-resistance in LAD is still unknown. This study aimed to observe the expression of ANRIL in LAD, evaluate its biological role in the resistance of LAD cells to paclitaxel and explore the apoptosis role in the ANRIL associated mechanism. Our results showed that ANRIL functioning as a potential oncogene was up-regulated in LAD, and promoted the acquisition of chemo-resistance in paclitaxel partly through the mitochondrial pathway by modulating the expression of apoptosis-related protein cleaved-PARP and Bcl-2. These findings might improve LAD patients' paclitaxel treatment and made ANRIL to be a new target for paclitaxel-based chemotherapy in LAD.

## INTRODUCTION

Lung cancer, with the first morbidity and the second death rate, is one of the common cancers occurred in both males and females in the world. Lung adenocarcinoma (LAD), as a typical type of non-small cell lung cancer, is at locally advanced or metastatic stage when diagnosis, which made patients had no time to do the early detection or treatment [1, 2]. Although tremendously therapeutic strategies have been improved in recent years, the five-year over-all survival rate of LAD patients is still dismal [3]. Paclitaxel is one of the most effective plant-derived anti-cancer drugs in LAD chemotherapy by causing cell cycle arrest in G2/M phase and cell apoptosis [4]. However, chemo-resistance remains a major impediment to clinical application of this drug.

Long non-coding RNAs (lncRNAs) are a family of non-protein-coding RNAs, which have been identified as oncogenes or tumor suppressors that are involved in a variety of diseases, including cancer [5–7]. ANRIL, also known as CDKN2B antisense RNA1, was originally identified in the familial melanoma patients. It is located within the CDKN2B-CDKN2A gene cluster at chromosome 9p21 [8, 9]. Since its identification, accumulating studies have showed that ANRIL may function as an oncogene and act as key players in many cancers, also in the cardiovascular disease [9–11]. However, the expression of ANRIL and its functional mechanisms in paclitaxel- resistant LAD are still ambiguous.

In the present study, we comprehensively investigated the function of ANRIL in paclitaxel-resistant LAD cells. Our results showed that ANRIL upregulated in LAD might promote the acquisition of chemo-resistance in paclitaxel.

## RESULTS

### ANRIL expression is increased in paclitaxel-resistant A549 cells

**Figure 1:**
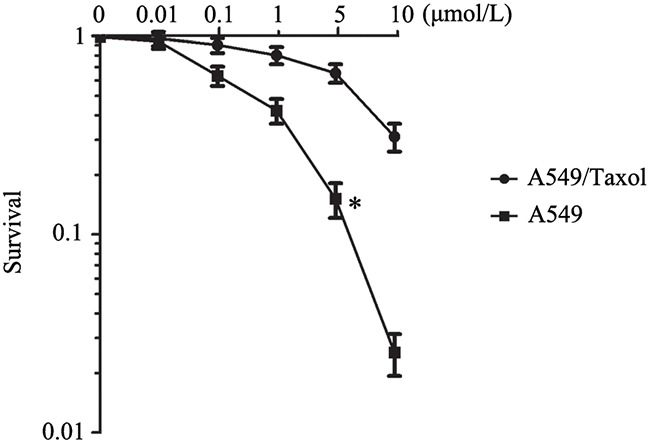
The establishment of paclitaxel resistance cell line A549/Taxol * *P* < 0.05 representsthe sensitivity of A549/Taxol to paclitaxel was greatly reduced, and the cells survival rate was increased significantly.

**Figure 2:**
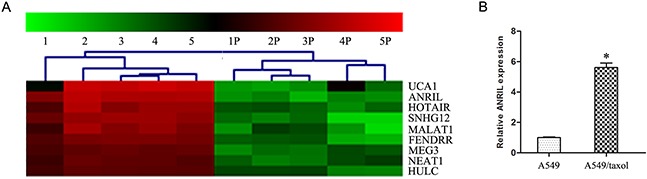
ANRIL is up-regulated in A549/Taxol cells **(A)** Representativemicroarray analysis of ANRILin A549/Taxol and parental A549 cells. **(B)** qRT-PCR assay of ANRILexpression level in A549/Taxol and parental A549 cells. * *P* < 0.05.

To identify the paclitaxel resistance associated lncRNAs in LAD A549 cells, we first successfully constructed the A549 paclitaxel resistant cell line A549/Taxol. As shown in Figure 1, the IC_50_ of A549/Taxol cells to paclitaxel was much higher than that in the A549 cells, especially in the 5 μmol/L paclitaxel treatment group (*P* < 0.05). Then we performed LncRNA microarray analysis between A549/Taxol and parental A549 cells. The result showed that ANRIL had an over ten-fold up-regulation in the A549/Taxol cells than that in parental A549 cells (Figure 2A). Following qRT-PCR results verified that a 5.6-fold mRNA expression level of ANRIL was observed in A549/Taxol when compared with the parental A549 cells (*P* < 0.001) (Figure 2B). This result suggests that ANRIL play a key role in the chemotherapy of LAD.

### ANRIL is an independent prognostic biomarker for LAD patients

**Figure 3:**
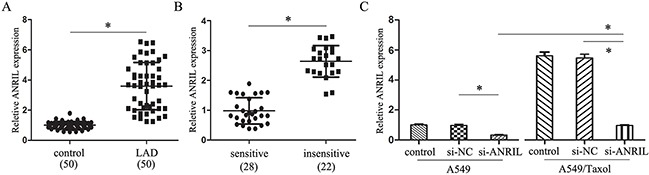
ANRIL inhibits the paclitaxel sensitivity of LAD **(A)** qRT-PCR assay of ANRILexpression level in LAD tissues (n = 50). **(B)** qRT-PCR assay of ANRILexpression level in LAD patients insensitive (n = 22) and sensitive (n = 28) to paclitaxel. **(C)** qRT-PCR assay of ANRILexpression level in A549/Taxol and parental A549 cells transfected with smart silencers. * *P* < 0.05.

We next investigated the ANRIL expression level in the LAD tissues by qRT-PCR, the results showed that the expression of ANRIL in LAD tissues was significantly higher than that in the adjacent normal tissues (Figure 3A). Clinical characterize analysis showed that increased ANRIL expression levels were positively correlated with poor differentiation grade (*P* = 0.040) and advanced pathologic stage (*P* < 0.001). However, ANRIL expression was not associated with other parameters such as gender (*P* = 0.550) and tumor size (*P* = 0.91) in LAD (Table 1). The Kaplan–Meier survival analysis showed that patients with higher ANRIL expression had a significantly lower survival time than patients with lower ANRIL expression (data not shown), which had the same tendency with Feng-qi Nie's research in 2015 [12]. Meanwhile, patients with higher expression of ANRIL had a significantly worse prognosis by a univariate Cox proportional hazards regression analysis of disease free survival (HR = 0.6957, 95% CI: 0.1705 to 1.221; *P* = 0.039). Together, we got the information that ANRIL was an independent prognostic biomarker for LAD patients, no matter them accepted paclitaxel associated chemotherapy or not.

**Table 1:** Univariate analysis the association between ANRIL expression and clinicopathological factors in 50 LAD patients

Factors		n	$\bar{\text{x}}\pm \text{s}$	*P*
Sex	Male	33	3.71 ± 0.16	0.550
	Female	17	3.68 ± 0.18	
size(cm)	≤3.0	19	3.65 ± 0.19	0.91
	3.0-6.0	24	3.74 ± 0.21	
	≥6.0	7	3.70 ± 0.20	
Differentiation	well/moderately	21	3.08 ± 0.14	0.040*
	poorly	29	4.15 ± 0.22	
T stasge	T1	14	3.12 ± 0.15	<0.001*
	T2	19	3.68 ± 0.23	
	T3	17	4.19 ± 0.26	
N stage	N0	13	3.04 ± 0.19	<0.001*
	N1	26	3.81± 0.22	
	N2	11	4.22 ± 0.24	
M stage	M0	42	3.57 ± 0.16	<0.001*

### ANRIL inhibits the paclitaxel sensitivity of LAD

To detect the effect of ANRIL gene in paclitaxel resistance, we divided the LAD patients' samples into two groups (+) (−) according to the sensitivity to paclitaxel or together with other chemotherapeutic drugs. By analysis with qRT-PCR, we found that the expression of ANRIL in (+) groups was much lower than that in the (−) group (Figure 3B). Also, ANRIL expression was much higher in the A549/Taxol transfected with si-ANRIL than that in the A549 transfection cells (Figure 3C). These results revealed that ANRIL up-regulation in LAD could inhibit the sensitivity to paclitaxel.

### ANRIL promotes the malignant behavior of A549/Taxol cells

**Figure 4:**
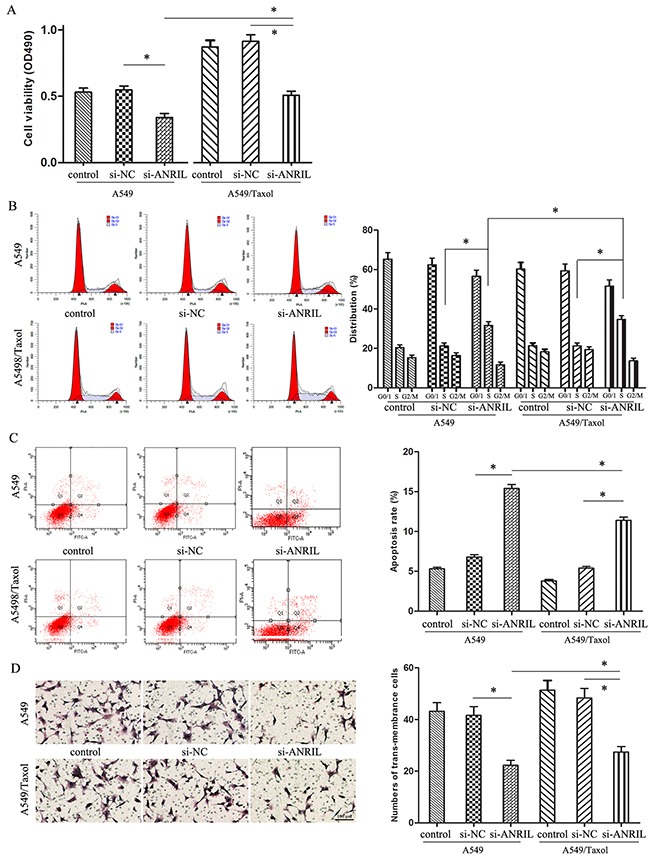
The effect of ANRIL on A549/Taxol cells biological behavior **(A)** MTT assay of A549/Taxol cell proliferation ability. **(B)** Flow cytometry analysis of cell cycle distribution in A549/Taxol cells. **(C)** Flow cytometry analysis of apoptosis in A549/Taxol cells. **(D)** Transwell chamber detecting the invasion ability of A549/Taxol cells. (Magnification: 200 ×). * *P* < 0.05.

As for the important role of ANRIL in LAD paclitaxel associated chemo-resistance, we further investigated the biological role of ANRIL in A549/Taxol cells. As shown in Figure 4A, the cell proliferation ability of A549/Taxol cells transfected with si-ANRIL was much higher than that in A549 cells with the same transfection (*P* < 0.05). From the DNA contents analysis, we found that A549/Taxol cells transfected with si-ANRIL had a significant S phase block, when compared with that in the A549 cells with the same transfection (*P* < 0.05) (Figure 4B). These results revealed that ANRIL participates in the cell proliferation of A549/Taxol cells. Then, flow cytometry assay showed that the apoptosis rate of cells were much lower in the A549/Taxol cells transfected with ANRIL than that in the A549 cells with the same transfection (*P* < 0.05) (Figure 4C), which indicated that ANRIL could reduce the paclitaxel associated resistance through inhibiting the cells apoptosis. Finally, we found that the trans-membrane cells were much more in the A549/Taxol cells transfected with ANRIL than that in the A549 cells with the same transfection (*P* < 0.05) (Figure 4D). Together, we got a conclusion that ANRIL could promote the malignant behavior and enhance the paclitaxel resistance of A549/Taxol cells.

### ANRIL decreases Bcl-2 expression and increases PARP expression

**Figure 5:**
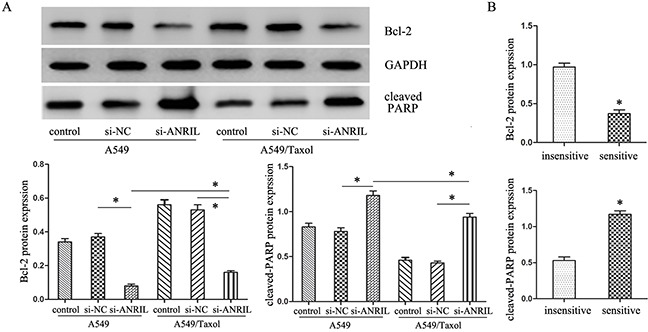
The expression of apoptosis-related protein cleaved-PARP and Bcl-2 **(A)** Western Blot analysis of cleaved-PARP and Bcl-2 protein expression levels after ANRIL smart silencer transfection. * *P* < 0.05. **(B)** Western Blot analysis of cleaved-PARP and Bcl-2 protein expression levels in LAD patients. * *P* < 0.05.

In order to investigate the mechanism of ANRIL in LAD A549/Taxol cells, we next predicted the probability RNA binding protein of ANRIL gene with the software StarBase, and found that Bcl-2 might be a target protein. Together with the published references, we chosen Bcl-2 associated apoptosis signal proteins as targets to highlight the apoptosis role in the ANRIL associated malignant behavior and paclitaxel resistance. Western blot assay showed that ANRIL could significantly decrease Bcl-2 expression and increase PARP expression no matter in the A549/Taxol cells or parental A549 cells. But A549/Taxol cells transfected with si-ANRIL had an increased Bcl-2 expression with a decrease PARP expression when compared to the parental A549 cells (P < 0.05) (Figure 5A). The same results could be seen in Figure 5B, Bcl-2 expression was increased while the PARP expression was decreased in paclitaxel sensitive (+) tissues compared with those in paclitaxel insensitive (−) tissues (P < 0.05). These results indicated that the abnormal expression of ANRIL in the advanced LAD patients was correlated with the response of patients to paclitaxel based chemotherapy partly through PARP and Bcl-2 modulating mitochondrial pathway.

## DISCUSSION

As we all known, LAD is got the attracted attention because its resistance to chemotherapy, while paclitaxel is a first-line chemotherapy drug for it. Altered expression of lncRNAs may provide novel therapeutic opportunities to cancer treatment and drug resistance [13–15]. However, the roles of lncRNAs in LAD carcinogenesis are not well understood. Therefore, exploring the paclitaxel chemotherapy associated lncRNAs and investigating the mechanisms by which lncRNAs function in LAD might facilitate the development of novel therapeutic targets that improve patients' prognosis.

This study observed the expression of ANRIL in LAD and evaluated its clinical significance in the resistance of LAD cells to paclitaxel. The finding showed that ANRIL was increased expression in LAD, especially in the LAD sensitive to paclitaxel or together with other chemotherapeutic drugs. By the bioinformatics analysis, the results revealed the expression of ANRIL was associated with the TNM stage and differentiation grade no matter patients were sensitive or insensitive to the paclitaxel associated chemotherapy. These data indicated that ANRIL involved in the process of paclitaxel associated chemo-resistance, could be an independent prognosis factor for LAD patients.

Although recently numerous evidences had showed that some abnormal expression lncRNAs might affect LAD chemo-resistance and most of them could be used as biomarkers and therapy targets [14, 16–18]. However, the potential function of ANRIL contributed paclitaxel chemo-resistance in LAD is still unknown. Till now, there were discordant opinions about the function of ANRIL in LAD [9] [19–21]. In order to verify whether ANRIL function as an oncogene or a tumor suppressor in the process of paclitaxel chemo-resistance in LAD, we further explored the biological role of ANRIL in LAD A549/Taxol cells by using MTT assay, apoptosis assay and transwell chamber assay. Our data further support the importance of ANRIL in LAD that ANRIL gene functions as an oncogene could promote the malignant behavior of A549/Taxol cells and enhance the resistance of paclitaxel.

At present, some lncRNAs like AK126698 and HOTAIR were considered to regulate the chemo-resistance of NSCLC cells [22–24]. Our study revealed that ANRIL also contributed to the chemo-resistance of LAD. However, the general problems made the underlying mechanisms are less well documented. Paclitaxel, as a standard front-line natural antitumor agent for LAD,, can induce up-regulation of the pro-apoptotic proteins Bax and Bak, as well as down-regulation and inactivation of the anti-apoptotic protein, Bcl-2 [25–27]. In this study, we chosen Bcl-2 associated apoptosis signal proteins as targets to highlight the apoptosis role in the ANRIL associated malignant behavior and paclitaxel resistance. Our results revealed that the abnormal expression of ANRIL in advanced LAD patients is correlated with the patients' response to paclitaxel based chemotherapy partly through modulating the expression of apoptosis-related protein cleaved-PARP and Bcl-2. Future study should investigate the detail molecular mechanisms underlying the ANRIL in the chemo-resistance of LAD.

## MATERIALS AND METHODS

### Paclitaxel resistant cell line A549/Taxol construction and cell culture

The human LAD A549 cells were stored by our lab and were cultured in RPMI 1640 medium supplemented with 10 % fetal bovine serum and cultured at 37°C as common. The paclitaxel-resistant A549/Taxol cells were constructed by continuous increasing co-culture with 0.1 μmol/L paclitaxel for one month, and then co-culture with medium containing 5 μmol/L paclitaxel to maintain the paclitaxel resistance. All cells in exponentially growing phase were used in the following experiments.

### Arraystar LncRNA array

Total RNA was extracted by the TRIzol® Reagent from A549/Taxol cells and parental A549 cells according to the operation manual. Then the total RNA was cleaned-up by the RNasey Mini Kit (p/n 74104, Qiagen, Ger). Human 8 × 60K LncRNA expression array provide by KangchengBio Corporation (Shanghai, China) was used for the detection of the lncRNAs/mRNAs expression profiling in the cells. In details, the cleaned RNA was labeled with Quick Amp Labeling Kit One-Color (p/n 5190-0442, Agilent, USA), purified with RNeasy Mini Kit. Then the labeled cRNA QC was assessed by NanoDrop ND-1000, following by hybridized with Agilent Gene Expression Hybridization Kit (p/n 5188-5242, Agilent, USA). After exhaustive washing, the arrays were scanned and the data were extracted by using Agilent Feature Extraction Software.

### Patients and tissue samples

Total paired LAD tissues and adjacent normal tissues (n = 50) were obtained from advanced LAD patients who underwent paclitaxel or combination with other chemotherapy for a maximum of four cycles from the Affiliated Shengjing Hospital of China Medical University between Feb 2010 and Jun 2013. These tissues were divided into two groups, one group (+) (n = 28) was complete or partial sensitive to the paclitaxel or together with other chemotherapeutic drugs, another group (−) (n = 22) was insensitive to the paclitaxel or together with other chemotherapeutic drugs. All the patients signed informed consent and the studies had got the approvement of the Ethics Committees. Tissue samples were immediately frozen in liquid nitrogen and stored at -80°C until use.

### qRT-PCR

Total RNA extraction and cDNA synthesis were finished as above. Then the RNA expression level was quantified with SYBR (# 4367659, Applied Biosystems, USA) by the 2^−ΔΔCt^ method through the 7500 Real-Time PCR System (Applied Biosystems, USA) when normalized to the expression of GAPDH.

### siRNA synthesis and cells transfection

The human ANRIL smart silencers were synthesized by the Ribobio Corporation (Guangzhou, China). The si-ANRIL or si-NC was transfected into the human LAD cell lines A549 and A549/Taxol by using Lipofectamine 3000 (Invitrogen, USA) according to the manufacturer's protocol. After 48 h, the cells cultured with ANRIL smart silencers were collected for the ANRIL biological effect assays and A549 cells chemo-resistance detection.

### Cell proliferation assay

The IC_50_ and cell proliferation ability were detected by MTT assay. For the IC_50_, A549 cells were seeded and cultured in RPMI 1640 medium. After 24 h, the cells were co-cultured with different concentrations (0.01, 0.1, 1, 5, 10 μmol/L) of paclitaxel. After incubation for 48 h, MTT agent was added and the following procedures were the same as before [28]. For the cell proliferation ability assay, the cells were seed into 96-well plate after smart silencer transfected 48 h, the detection was done also as before.

### Flow cytometry assay

Cell cycle and cell apoptosis assays were detected by the Flow cytometry. The apoptosis detection was finished with Annexin V-FITC apoptosis detection kit (Biosea, China) 48 h after transfection and analyzed using CELLQuest software. Cells in the right lower quadrant were regarded as apoptosis. For the cell cycle assay, 1 × 10^6^ cells were collected and following fixed and stained with propidium iodide, the cell cycles were determined by the Flow Cytometry (FACScan, Becton Dickinson, USA) within 1 h.

### Transwell assay

The invasion ability of cells was determined by transwell chamber (Coster, USA) according to the manufacturer's protocol. In brief, the upper compartment was added a density of 2 × 10^5^ cells/well in 25 μl of serum free medium and the bottom chamber was added 0.5 ml supernatant of human NIH3T3. After incubation for the appropriate time at 37°C, the number of cells invaded to the lower chamber was stained with hematoxylin & eosin and counted.

### Western blot

Transfected cells were collected and lysed with RIPA buffer (Beyotime, Shangahi, China). Total 30 μg of protein lysates were added to the 10 % SDS-PAGE and then the proteins were transferred to a 0.22 μm PVDF membrane. After incubated with specific antibodies, signals were visualized using the infrared labeled antibodies through the Dual Color Infrared Laser Imaging System (Gene, HK, China) by normalized to the inference gene of GAPDH.

### Statistical analysis

Data are shown as means ± SD. All results were got from at least three independent experiments. Student's *t* test, ANOVA and Spearman's correlation analysis were performed with the SPSS 17.0 software for statistical analysis. *P* < 0.05 was considered to be statistic significane.
